# A Tandem Solar Biofuel Cell: Harnessing Energy from Light and Biofuels

**DOI:** 10.1002/anie.202012089

**Published:** 2020-11-24

**Authors:** Marc Riedel, Soraya Höfs, Adrian Ruff, Wolfgang Schuhmann, Fred Lisdat

**Affiliations:** ^1^ Biosystems Technology Institute of Life Sciences and Biomedical Technologies Technical University of Applied Sciences Wildau Hochschulring 1 15745 Wildau Germany; ^2^ Analytical Chemistry—Center for Electrochemical Sciences (CES) Faculty of Chemistry and Biochemistry Ruhr-University Bochum Universitätstr. 150 44780 Bochum Germany; ^3^ PPG (Deutschland) Business Support GmbH EMEA Packaging Coatings Erlenbrunnenstr. 20 72411 Bodelshausen Germany

**Keywords:** biocatalysis, biofuel cells, energy harvesting, photocatalysis, photoelectrochemistry

## Abstract

We report on a photobioelectrochemical fuel cell consisting of a glucose‐oxidase‐modified BiFeO_3_ photobiocathode and a quantum‐dot‐sensitized inverse opal TiO_2_ photobioanode linked to FAD glucose dehydrogenase via a redox polymer. Both photobioelectrodes are driven by enzymatic glucose conversion. Whereas the photobioanode can collect electrons from sugar oxidation at rather low potential, the photobiocathode shows reduction currents at rather high potential. The electrodes can be arranged in a sandwich‐like manner due to the semi‐transparent nature of BiFeO_3_, which also guarantees a simultaneous excitation of the photobioanode when illuminated via the cathode side. This tandem cell can generate electricity under illumination and in the presence of glucose and provides an exceptionally high OCV of about 1 V. The developed semi‐artificial system has significant implications for the integration of biocatalysts in photoactive entities for bioenergetic purposes, and it opens up a new path toward generation of electricity from sunlight and (bio)fuels.

## Graphical abstract

A photobioelectrochemical tandem cell is presented in which two photoelectrodes have been functionally coupled with two biocatalysts for supplying the light‐driven reaction with charge carriers from glucose conversion. The cell allows the generation of electricity from biofuels and light with a high open‐circuit voltage of 1 V.

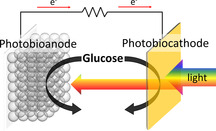

Interfacing biotic components with abiotic entities on electrodes has gained considerable interest for power generation, the production of fuels and chemicals, but also for sensing.[^1^, ^2^] Particularly, the coupling of photoactive materials with biocatalysts provides a promising strategy for the introduction of new catalytic features in solar‐driven signal chains, which are not feasible by each component alone.^[3]^

Besides the connection of the photoactive entity to the electrode, in particular, the efficient linkage of the enzyme is a key for the construction of high performance photoactive biohybrids. Often light‐driven signal chains are established via photoelectrochemical oxidation/reduction of enzymatic products and substrates,[^4^, ^5^, ^6^, ^7^] or mediators,[^8^, ^9^, ^10^, ^11^] while the direct electron transfer remains challenging.[^12^, ^13^] Recently, we and others have demonstrated that FAD glucose dehydrogenase^[14]^ and photosystem II[^15^, ^16^, ^17^] can be wired to photoanodes, resulting in an improved onset potential for glucose and H_2_O oxidation, respectively. If such a biohybrid photoanode is coupled to a light insensitive biocathode, electrons from biocatalytic oxidation can be transferred to a reductase reaction under illumination for the generation of electric energy with a high operational voltage,^[17]^ or the production of hydrogen^[15]^ and formate.^[16]^ Several photobiocathodes have been reported in literature, however, they often suffer from rather high overpotentials to generate a photocurrent or to drive an enzymatic reaction.[^18^, ^19^] Moreover, (photo‐)cathodes that mimic enzymatic reactions such as H^+^, H_2_O_2_ or O_2_ reduction are often significantly worse in terms of overpotential and conversion rate than their biocatalytic counterparts. For example, bioelectrocatalytic H_2_O_2_ reduction using horseradish peroxidase[^20^, ^21^] and decaheme cytochrome^[22]^ is found to occur several hundred mVs more positive than in most photoelectrocatalytic approaches.[^19^, ^23^, ^24^] Therefore, new strategies for the construction of photo(bio)cathodes are necessary to overcome energetic losses for light‐driven chemical reduction.

Besides the use of single photoactive electrodes, the combination photocathodes with photoanodes in a solar tandem cell format has become a focus of research aiming to improve solar yield and operating voltage.^[25]^ Construction of a solar tandem cell with two photobioelectrodes could set new benchmarks for the synthesis of chemicals, but can also provide a new way for the construction of photobiofuel cells, which combine energy production from light and (bio)fuels.

Here, we introduce a new concept for the generation of power from light and biofuels by combination of a glucose converting photobiocathode with a glucose‐powered photobioanode (Scheme 1). For this, a BiFeO_3_ photocathode with high photocatalytic activity towards H_2_O_2_ reduction has been coupled to glucose oxidase (GOx). The biocatalytic glucose turnover generates H_2_O_2_, which accepts excited electrons from the photocathode under concomitant amplification of the cathodic photocurrent. The final BiFeO_3_|GOx photobiocathode is coupled to a glucose converting photobioanode in a photobioelectrochemical tandem cell (PBTC) set‐up, enabling the generation of energy with high cell voltages under illumination and in the presence of biofuel.

**Scheme 1:**
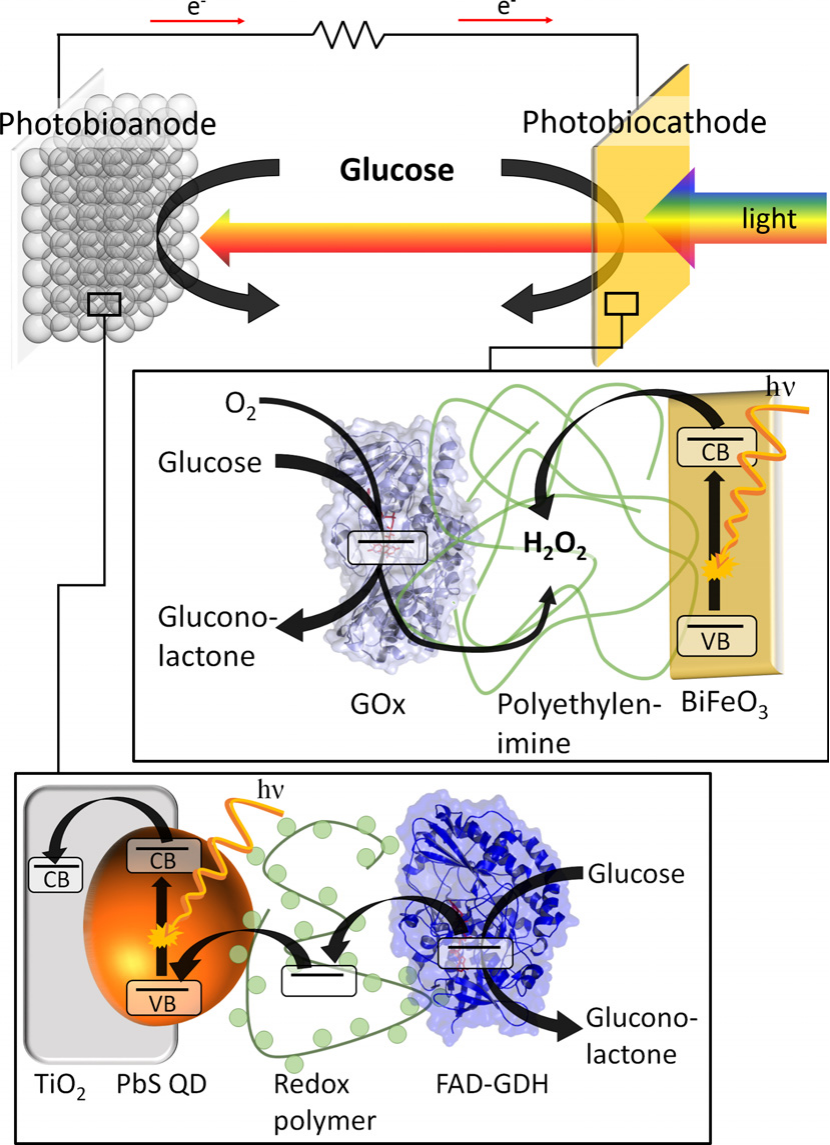
Illustration of the photobioelectrochemical tandem cell consisting of a BiFeO_3_|GOx photobiocathode and an IO‐TiO_2_|PbS|P_Os_|FAD‐GDH photobioanode, and the proposed electron transfer steps of the signal chain under illumination and in the presence of glucose. PBD ID GOx: 1CF3,^[30]^ PDB ID FAD‐GDH: 4YNT.^[31]^

As a starting point we have investigated BiFeO_3_ for its suitability as new semi‐transparent, light‐sensitive photobiocathode material due to its photoelectrochemical properties.[^26^, ^27^] BiFeO_3_ photocathodes have been prepared on fluorine doped tin oxide (FTO) slides by a spin coating approach using Bi(NO_3_)_3_ and Fe(NO_3_)_3_ with a ratio of 1:1 and subsequent sintering. The resulting BiFeO_3_ layer is yellowish colored and from the UV/Vis data an optical band gap of about 2.7 eV can be determined—in good agreement with a previous report (Supporting Information, Figures S1 and S2).[^28^, ^29^] SEM images reveal the formation of a rather homogeneous porous surface with no large agglomerates (Figure 1 A), preventing larger light scattering effects and thus providing enough transparency (about 65 % at 550 nm) to allow excitation of a photobioanode with a smaller band gap (i.e. larger wavelength excitation range).

**Figure 1:**
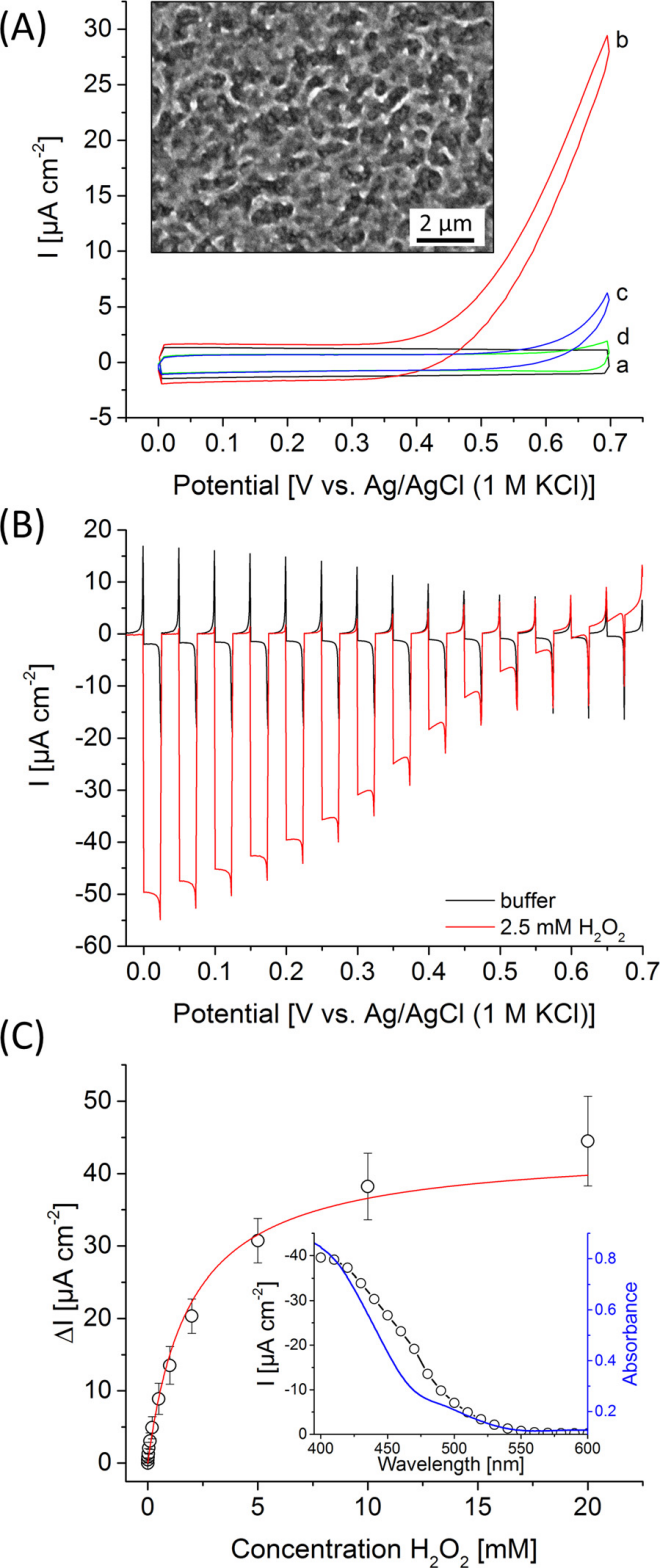
A) Cyclic voltammograms of pure FTO slides (a,b) and FTO|BiFeO_3_ electrodes (c,d) in the presence (b,c) and absence (a,d) of 2.5 mM H_2_O_2_ in the dark (100 mV s^−1^). Inset: SEM image of a BiFeO_3_ electrode with a 10 000‐fold magnification. B) Chopped‐light voltammetry of a BiFeO_3_ electrode in the presence and absence of 2.5 mM H_2_O_2_ (100 mW cm^−2^; 10 mV s^−1^). C) Photocurrent density change Δ*I* of a BiFeO_3_ electrode before and after addition of different H_2_O_2_ concentrations (100 mW cm^−2^; 0.2 V vs. Ag/AgCl, 1 M KCl). Inset: wavelength‐dependent photocurrent response (black points) and UV/Vis spectrum (blue line) of a BiFeO_3_ electrode (0.2 V vs. Ag/AgCl, 1 M KCl).

Photocatalytic H_2_O_2_ reduction is often counterbalanced by disturbing H_2_O_2_ oxidation processes occurring at the base electrode, thus reducing the performance in terms of potential behavior. To study this effect basic electrochemical investigations have been performed in the dark with pure FTO and FTO|BiFeO_3_ electrodes in the presence and absence of H_2_O_2_. While pure FTO electrodes show strong H_2_O_2_ oxidation starting at ≈0.3 V vs. Ag/AgCl (1 M KCl), anodic processes can drastically be reduced by modification with BiFeO_3_ (Figure 1 A). This indicates that BiFeO_3_ passivates the FTO and interferes with the penetration of H_2_O_2_ to the underlying FTO and considerably minimize parasitic H_2_O_2_ oxidation. This surface passivation effect has also been verified by impedance experiments in the presence of ferri/ferrocyanide, resulting in a 40‐fold enhancement of the charge transfer resistance from 20 kΩ for FTO to about 800 kΩ for BiFeO_3_ electrodes (Figure S3).

The BiFeO_3_ electrode has been first investigated photoelectrochemically via chopped light voltammetry. Figure 1 B displays the formation of a cathodic photocurrent over the whole investigated potential range in buffer. In the presence of H_2_O_2_ this reduction current is significantly enhanced. For instance, at 0 V vs. Ag/AgCl (1 M KCl) the current is amplified 15 times (−2.8±1.9 μA cm^−2^ to −42.3±8.7 μA cm^−2^), giving rise to an external quantum efficiency (EQE) of 0.1±0.02 %. In contrast, pure FTO slides exhibit no photoelectrochemical response (Figure S4). An onset potential of 0.63±0.016 V has been obtained for H_2_O_2_ reduction, which is to the best of our knowledge more than 0.2 V more positive than previous PEC approaches[^19^, ^23^, ^24^] and also slightly exceeds the onset potential of light‐independent bioelectrocatalytic approaches using enzymes.[^20^, ^21^, ^22^] This highlights the outstanding photoelectrocatalytic activity found for the BiFeO_3_ electrodes as peroxidase mimics. Moreover, wavelength resolved measurements demonstrate that the photocurrent response of the BiFeO_3_ nicely matches with the absorbance features of the structures, showing reasonable photocurrents below 550 nm and proves BiFeO_3_ as the origin of the photosignal (Figure 1 C).

The BiFeO_3_ photocathodes show a typical saturation‐type concentration behavior for H_2_O_2_ reduction giving first signals at 5 μM up to concentrations of 20 mM H_2_O_2_ at a rather positive working potential of 0.2 V vs. Ag/AgCl (1 M KCl) (Figure 1 C; Figure S5). This makes BiFeO_3_ photocathodes interesting candidates for sensing and for the combination with H_2_O_2_ producing enzymes. Furthermore, a reasonable stability of about 80 % of the initial signal has been found for the light‐directed H_2_O_2_ reduction after 15 min pulsed illumination (Figure S6).

Based on these findings, we have combined the photocatalytic activity of the BiFeO_3_ electrode towards H_2_O_2_ with the biocatalytic features of GOx for the construction of a glucose‐driven photobiocathode. The signal chain is initiated by the biocatalytic turnover of glucose to gluconic acid and concomitant generation of H_2_O_2_, which reacts at the BiFeO_3_ under illumination and results in enhanced cathodic photocurrents. To achieve a high amount of biocatalysts in front of the electrode, the enzyme has been embedded into polyethylenimine and stabilized via crosslinking. SEM investigations reveal a rather high surface roughness with a height of about 1.1 μm (Figure S7). Here, a GOx activity of at least 0.8±0.09 U has been integrated into this polymeric network (for details see Figure S8). The final BiFeO_3_|GOx electrode has been studied with respect to H_2_O_2_ sensitivity. It provides a similar signal response to a defined H_2_O_2_ concentration as an unmodified electrode, indicating that the enzyme modification does not affect the catalytic electrode activity (Figure S9). Furthermore, the BiFeO_3_|GOx electrodes have been investigated via chopped light voltammetry in the absence and presence of glucose. As depicted in Figure 2, the cathodic photocurrent is enhanced over the whole potential range after addition of 10 mM glucose (ΔI=14.6±2.8 μA cm^−2^, 0 mV vs. Ag/AgCl (1 M KCl)), while electrodes without GOx show no signal increase (Figure S10). This clearly confirms the functionality of the constructed signal chain as illustrated in Scheme 1.

**Figure 2:**
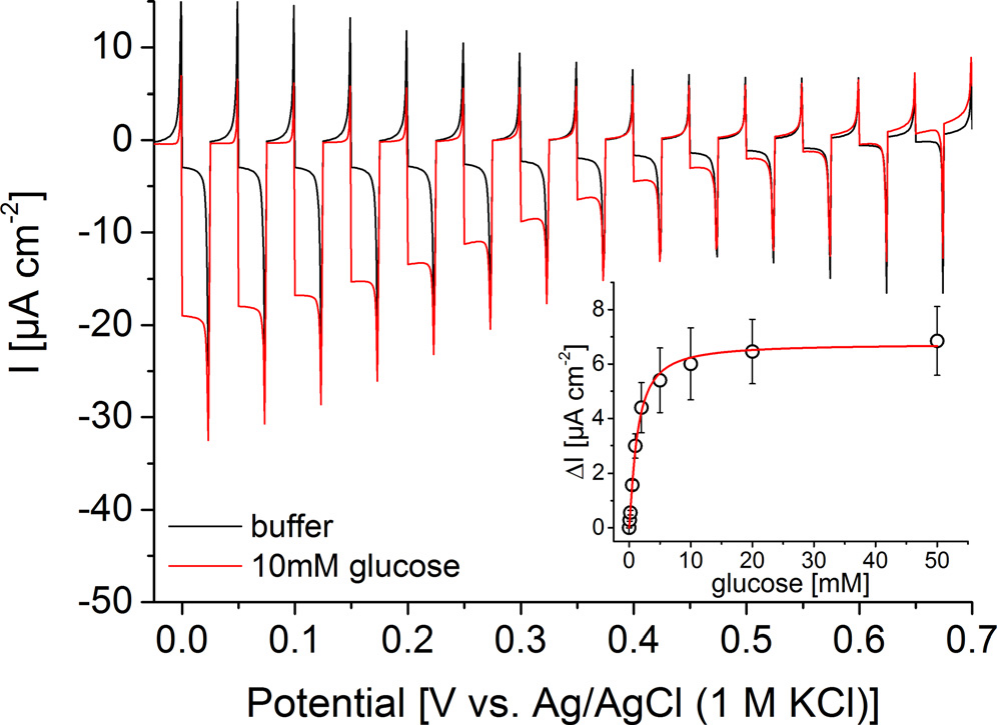
Chopped‐light voltammetry of a BiFeO_3_|GOx electrode in the presence and absence of 10 mM glucose (100 mW cm^−2^; potential vs. Ag/AgCl, 1 M KCl; 10 mV s^−1^). Inset: photocurrent density change Δ*I* of a BiFeO_3_|GOx electrode before and after addition of different glucose concentrations (100 mW cm^−2^; 0.2 V vs. Ag/AgCl, 1 M KCl).

The maximum current of the BiFeO_3_|GOx electrode under glucose‐saturated conditions corresponds to a H_2_O_2_ concentration of about 300 μM produced by the immobilized GOx. This concentration is comparable to the oxygen content in solution and suggests that the electrode performance is mainly restricted by the low oxygen availability in solution. This is a typical problem of oxygen‐reducing electrodes, but can be circumvented by improving the oxygen transport to the electrode.^[32]^

The onset potential has been determined to be 0.602±0.007 V vs. Ag/AgCl (1 M KCl), which demonstrates the efficient coupling of enzymatic H_2_O_2_ production and light‐triggered turnover. The concentration dependency of the BiFeO_3_|GOx electrodes has been determined at a bias of 0.2 V vs. Ag/AgCl by successively increasing the glucose concentration (Figure 2). Here, a signal response has been observed starting at 100 μM glucose and leveling off at about 50 mM. An apparent K_M_ value of 1.3±0.1 mM can be determined. The results illustrate the good conditions found for the construction of the photobiocathode. Glucose conversion can be combined with current generation at rather positive potential.

Subsequently, the photobiocathode is coupled to an inverse opal (IO)‐TiO_2_ photobioanode modified with PbS quantum dots (QDs), redox polymer (P_Os_) and FAD glucose dehydrogenase (FAD‐GDH) as reported by us before (for a SEM image see Figure S11).^[14]^ While PbS QDs act as photoactive entity and introduce visible light sensitivity into the photoanode, the redox polymer mediates the electron transfer from the enzyme towards the QDs to the IO‐TiO_2_ architecture in the presence of glucose under illumination as depicted in Scheme 1. The photobioanode gives rise to maximum anodic photocurrents of 151±29 μA cm^−2^ at 0 V vs. Ag/AgCl (1 M KCl) in the presence of glucose (Figure 3 A). An open circuit potential of −0.463±0.004 V vs. Ag/AgCl (1 M KCl) is formed under illumination, allowing electron extraction from biocatalytic glucose oxidation at quite negative bias (Figure 3 B). We have chosen PbS as photoactive entity due to its small band gap,^[14]^ this should allow excitation at higher wavelengths as compared to the BiFeO_3_ material.

**Figure 3:**
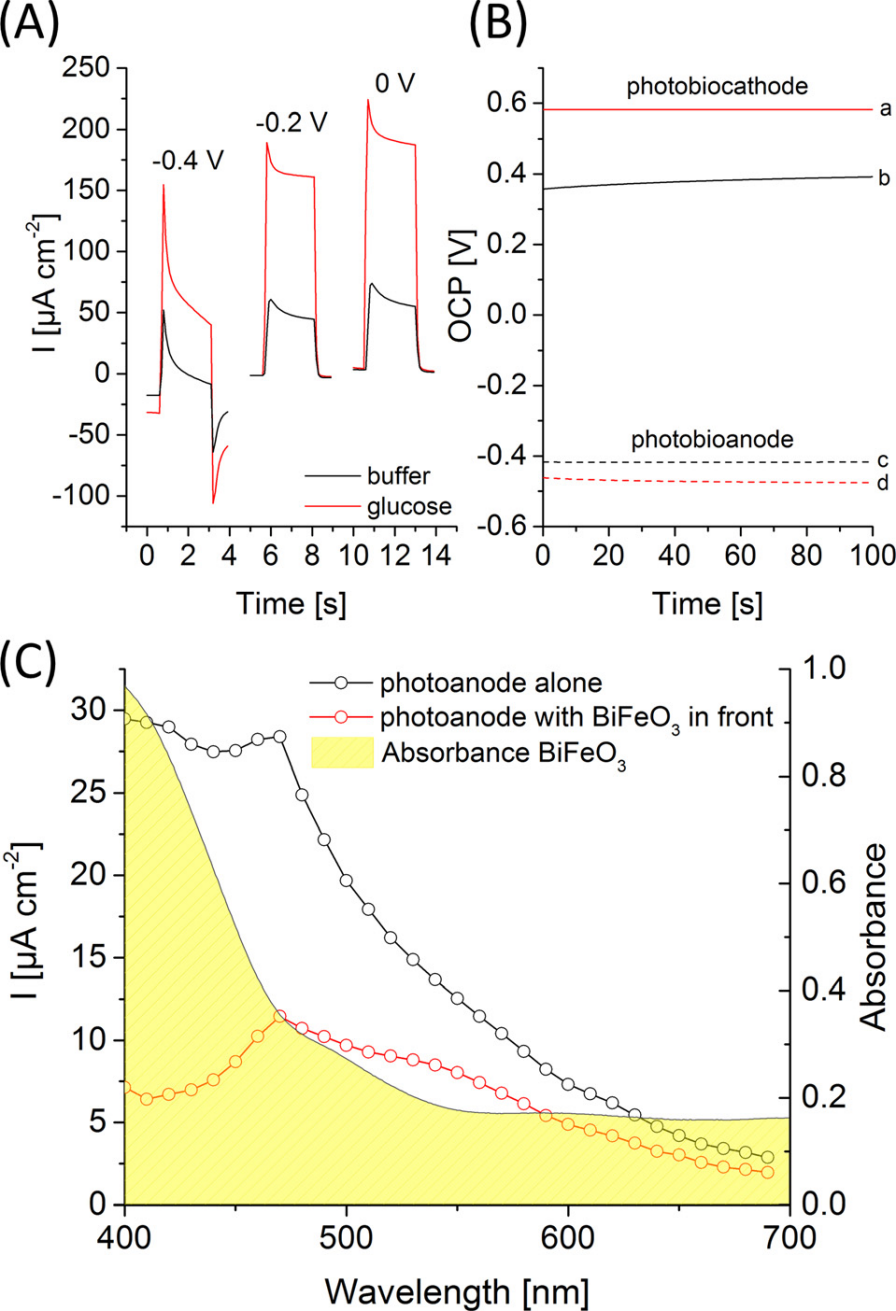
A) Photocurrent response of an IO‐TiO_2_|PbS|P_Os_|FAD‐GDH electrode in the presence and absence of 100 mM glucose at different potentials (100 mW cm^−2^; potential vs. Ag/AgCl, 1 M KCl). B) Potentiometric measurement of the photobiocathode (a,b) and the photobioanode (c,d) in the presence (a,d) and absence (b,c) of glucose under illumination (100 mW cm^−2^; OCP vs. Ag/AgCl, 1 M KCl). C) Wavelength‐dependent photocurrent of an IO‐TiO_2_|PbS|P_Os_|FAD‐GDH electrode with unimpeded illumination (black curve) and by illumination through the BiFeO_3_ (red curve) material (0 V vs. Ag/AgCl, 1 M KCl). Additionally, the UV/Vis spectrum of BiFeO_3_ is shown.

To scrutinize the influence of the BiFeO_3_ absorbance on the signal generation at the photobioanode, wavelength resolved measurements are performed with and without BiFeO_3_ in front of the electrode (Figure 3 C). As expected, the photoresponse of the photobioanode is diminished, particularly at wavelengths below 550 nm where BiFeO_3_ is absorbing. But even under these conditions, a reasonable part of the light can pass the cathode for the excitation of the photobioanode, resulting in pronounced photocurrents of 83.5±15.2 μA cm^−2^ under white light illumination (Figure S12). Since this is still larger than the photocurrent generation at the photobiocathode, advantageous conditions are provided for an on top arrangement of both electrodes (Figure S13). Thus, the optical properties of the photobiocathode nicely fit to the excitation range of the photobioanode, so that both can be simultaneously excited.

Consequently, for the construction of the PBTC, the BiFeO_3_|GOx electrode and the IO‐TiO_2_|PbS|P_Os_|FAD‐GDH electrode have been arranged opposite each other so that the light passes the photobiocathode first and then reaches the photobioanode (Scheme 1). The advantage of this arrangement is that the necessary space for the cell is reduced and this allows a higher energy yield per area (or volume). *I*–*V* curves have been performed in the presence and absence of glucose under illumination in order to investigate the power generation of the cell. As illustrated in Figure 4, a quite large open cell voltage (OCV) of 0.995±0.006 V and a photocurrent of up to 23.9±3.5 μA cm^−2^ has been obtained with glucose in solution. A maximum power density of 8.1±1.1 μW cm^−2^ can be determined at a cell potential of 0.55±0.02 V. The OCV correlates well with the individual potentials of the photobiocathode and photobioanode (Figure 3 B) under illumination, while the photocurrent of the cell is limited by the cathodic reaction. In the absence of glucose only small photocurrents of 1.8±0.65 μA cm^−2^ are observed. This clearly demonstrates that the biocatalytic glucose turnover is necessary to drive both, the photobiocathode and the photobioanode. The PBTC shows acceptable stability with glucose in solution and under illumination with high intensity reaching 70 % of the initial signal after 20 min operation (Figure S14). To the best of our knowledge, the results represent the first example for a PBTC, in which the power generation is realized by combination of two photobioelectrodes that can be arranged sandwich‐like saving space. While the photocurrent amplitude is currently modest, the open circuit voltage is higher as compared to classical light‐insensitive glucose biofuel cells.[^33^, ^34^, ^35^, ^36^, ^37^] However, since inorganic metal oxide and quantum dot‐based photoelectrodes already allow currents in the mA range,[^38^, ^39^] there is great potential for improvement for future biohybrid tandem solar cells, if the biotic/abiotic interface is adjusted.

**Figure 4:**
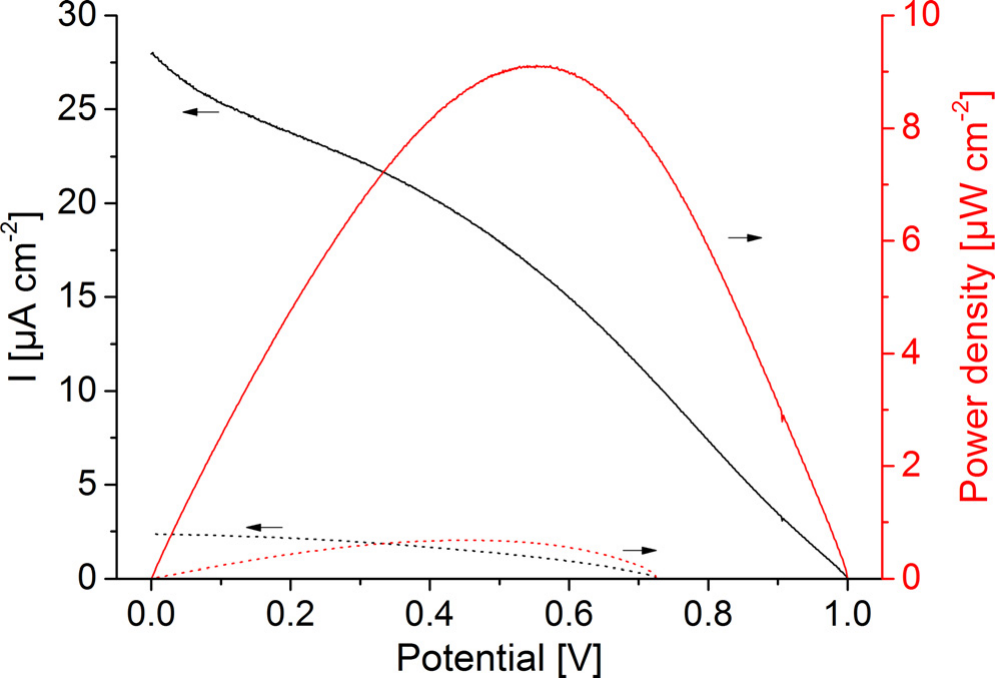
Current and power density of the photobioelectrochemical tandem cell, consisting of an BiFeO_3_|GOx photobiocathode and an IO‐TiO_2_|PbS|P_Os_|FAD‐GDH photobioanode in the presence (solid lines) and absence (dotted lines) of 100 mM glucose and under illumination. The tandem cell is arranged in a sandwich‐like manner, so that the photoexcitation of both electrodes is realized by lighting through the semi‐transparent photobiocathode (100 mW cm^−2^; 5 mV s^−1^).

In summary, we have demonstrated a proof‐of‐concept for a photobioelectrochemical tandem cell in which two photoelectrodes are functionally coupled with two biocatalysts for supplying a light‐driven reaction with charge carriers from glucose conversion. A BiFeO_3_|GOx electrode has been designed for the cathodic reaction, which is based on a high photoelectrocatalytic activity towards H_2_O_2_ and enzymatic H_2_O_2_ production in the presence of glucose, thereby giving access to first photocurrents at quite positive potentials of about 0.6 V vs. Ag/AgCl. The photobiocathode has been combined with a glucose‐converting IO‐TiO_2_|PbS|P_Os_ photoanode hosting FAD‐GDH in a sandwich‐like arrangement so that a reasonable photoexcitation of both photoactive electrodes is realized by illumination through the semi‐transparent photobiocathode. The biohybrid cell is capable of generating electricity under illumination and in the presence of one fuel molecule, reaching a high OCV of about 1 V. It is anticipated that this study will advance the development of biohybrid tandem cells for energy demands so that improvements in the photoelectrode construction and enzyme/semiconductor interface (e.g. by nanostructuring) will give access to more efficient systems with higher power output in future.

## Conflict of interest

The authors declare no conflict of interest.

## Supporting information

As a service to our authors and readers, this journal provides supporting information supplied by the authors. Such materials are peer reviewed and may be re‐organized for online delivery, but are not copy‐edited or typeset. Technical support issues arising from supporting information (other than missing files) should be addressed to the authors.

Supplementary
