# Supplementary material for: A Tandem Solar Biofuel Cell: Harnessing Energy from Light and Biofuels
Source: Angew Chem Int Ed Engl. 2020 Nov 24;60(4):2078–83. doi: 10.1002/anie.202012089 (PMC7894536; doi:10.1002/anie.202012089)
Supplement: Supplementary file 1 — Supplementary [file ANIE-60-2078-s001.pdf]

Angewandte Chemie
Eine Zeitschrift der Gesellschaft Deutscher Chemiker

Supporting Information

A Tandem Solar Biofuel Cell: Harnessing Energy from Light and Biofuels

Marc Riedel, Soraya Höfs, Adrian Ruff, Wolfgang Schuhmann, and Fred Lisdat**

anie_202012089_sm_miscellaneous_information.pdf

Experimental Procedures

Materials: 2-(N-Morpholino)ethanesulfonic acid (99.5%; MES), 2-methoxyethanol (99.98%), iron (III) nitrate nonahydrate (99.99%; $Fe(NO_3)_3$), fluorine-doped tin oxide-coated glass slides (7 Ω cm; FTO), lead(II) nitrate (99.999%; $Pb(NO_3)_2$), polyethylenimine solution (50% (w/v) in H_2O; PEI), poly(ethylene glycol) diglycidyl ether (Mn 500, PEGDGE), ammonium sulfide solution (20% in H_2O; $(NH_4)_2S$), 0.8 µm polystyrene latex beads (LB), titanium tetraisopropoxide (97%; TTIP) and glucose oxidase (GOx) from *Aspergillus niger* (207 U mg^{-1}) were purchased from Sigma-Aldrich. Bismuth(III) nitrate pentahydrate (>97%; $Bi(NO_3)_3$) was obtained from Alfa Aesar GmbH + Co. KG and D-glucose, dipotassium hydrogen phosphate and potassium dihydrogen phosphate were purchased from CARL ROTH GmbH + Co. KG. FAD-dependent glucose dehydrogenase from *Aspergillus sp.* has been obtained from SEKISUI CHEMICAL. Poly(1-vinylimidazole-*co*-allylamine)-Os(bipyridine)$_2$Cl-redox polymer (P_{Os}) has been synthesized as reported before.[1]

Construction of $BiFeO_3$|GOx Electrodes: FTO-coated glass slides were alternately cleaned with deionized water, isopropanol and acetone in an ultrasonication bath for 15 min each and dried at 120°C for 30 min. Subsequently, a mixture containing $Bi(NO_3)_3$ and $Fe(NO_3)_3$ precursors was spin coated onto the FTO slides as reported previously with some modifications.[2] In detail, 10 µl 0.5 M $Bi(NO_3)_3$ and 0.5 M $Fe(NO_3)_3$ in 2-methoxyethanol was drop-casted onto the FTO and spin coated after incubation for 10 s with 150 rps for 90 s. The modified electrodes were sintered at 550 °C under air for 3 h, resulting in the final $BiFeO_3$ electrodes.
For the construction of the $BiFeO_3$|GOx electrodes, 4µl of a mixture containing 5mg ml^{-1} GOx, 5mg ml^{-1} polyethylenimine and 0.2 mg ml^{-1} PEGDGE was drop-casted onto the electrodes and incubated for 2 h. The final electrodes were carefully rinsed two times with 1 ml buffer (50 mM potassium phosphate buffer pH 7) to remove unbound material.

Construction of IO-TiO_2|PbS|P_{Os}|FAD-GDH Electrodes: FTO-coated glass slides were cleaned as described above. IO-TiO_2 structures were constructed by a protocol established by us previously.[3] Briefly, a mixture of 100 mg ml^{-1} TTIP and 100 mg ml^{-1} latex beads with a diameter of 0.8 µm was prepared in isopropanol and deposited by a spin coating approach onto the FTO slides. Eight layers of the TTIP/LB mixture were spin coated at 80 rps in order to obtain an IO-TiO_2 structure with a thickness of about 10 µm. Subsequently, the electrodes were sintered at 450 °C under air for 2 h. After preparation of the IO-TiO_2 architecture PbS QDs were directly grown on the electrodes by a successive ionic layer adsorption and reaction (SILAR) approach following a previously reported protocol.[3] Therefore, the electrodes were alternately immersed four times in aqueous 20 mM $Pb(NO_3)_2$ and aqueous 20 mM $(NH_4)_2S$ solution for 1 min, respectively. In order to remove unbound precursors the electrodes were carefully rinsed with deionized water and ethanol between the deposition steps. The redox polymer and FAD-GDH were integrated into the final IO-TiO_2|PbS electrodes by a co-assembly approach, Therefore, 4 µl of a mixture containing 5 mg ml^{-1} P_{Os} and 5 mg ml^{-1} FAD-GDH (in 5 mM MES buffer pH 7) was deposited onto the electrodes in the dark. After 15 min the electrodes were carefully rinsed with buffer (50 mM potassium phosphate buffer pH7) to remove unbound material.

Construction of the Photobioelectrochemical Tandem Cell: For the construction of the PBTC the $BiFeO_3$|GOx photobiocathode and the IO-TiO_2|PbS|P_{Os}|FAD-GDH photobioanode were arranged opposite each other. The cell was filled with 50 mM potassium phosphate buffer pH7 with or without 100 mM glucose and illumination was performed through the cathode side.

Photoelectrochemical and Electrochemical Experiments: Photoelectrochemical and electrochemical measurements were performed with an integrated photoelectrochemical workstation from Zahner, containing a potentiostat for the light control and a second potentiostat (Zennium) for the electrochemical control. In the case of an illumination, a white light source (410−800 nm) was used unless stated otherwise. Wavelength-resolved measurements were performed with a monochromator (Polychrome V, Till Photonics) with a bandwidth of 15 nm. The (photo)electrochemical characterization of the photobioanode and the photobiocathode was performed in a homemade electrochemical cell with a three-electrode arrangement, consisting of a working electrode (photobioanode or photobiocathode), a platinum wire as counter electrode, and an Ag/AgCl, 1 M KCl, reference electrode.
For the cell experiments, linear sweep polarization curves were recorded from the open circuit voltage (OCV) to 0 V with the photobioanode connected as working electrode and the photobiocathode as combined reference/counter electrode. All measurements were performed in 50 mM potassium phosphate buffer pH7.

SEM Experiments: SEM experiments were performed with a JSM-6510 from JEOL at an acceleration voltage of 30 kV and 15 kV for pure $BiFeO_3$ and $BiFeO_3$|GOx electrodes, respectively.

Results and Discussion

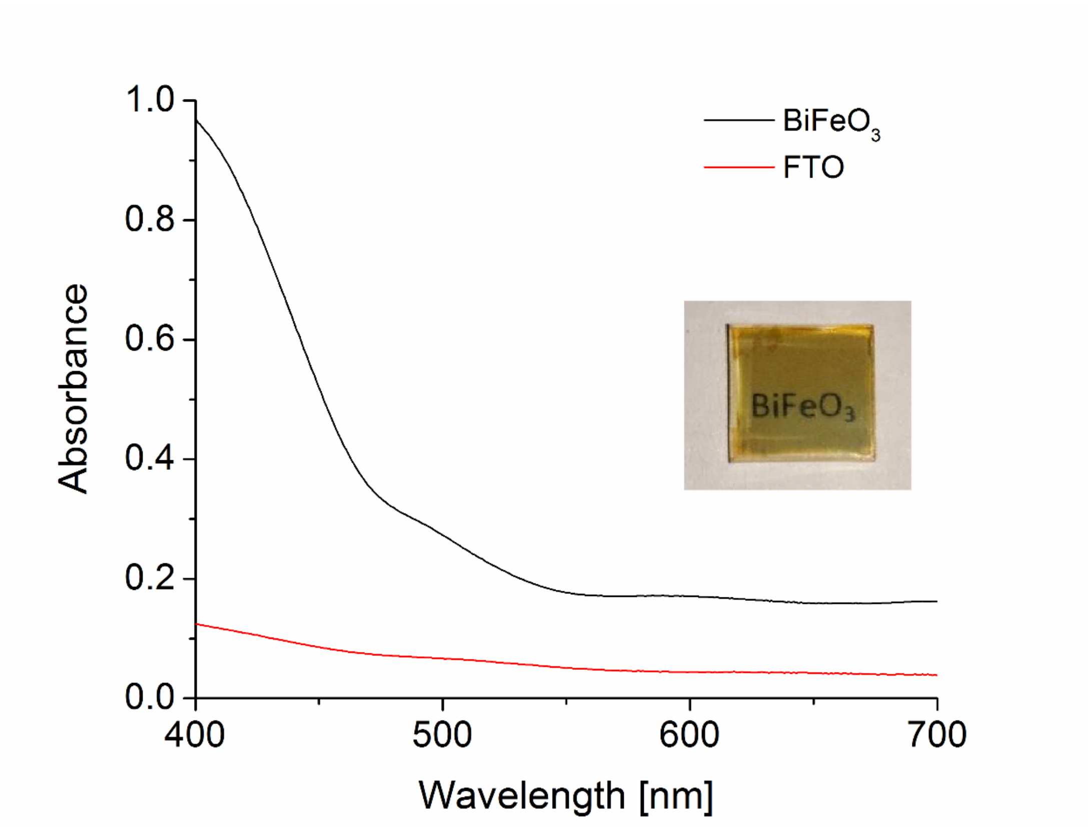

Figure S1 UV/Vis spectra of an FTO slide before and after deposition of $BiFeO_3$ in water. Inset: Photographic image of the $BiFeO_3$ electrode.

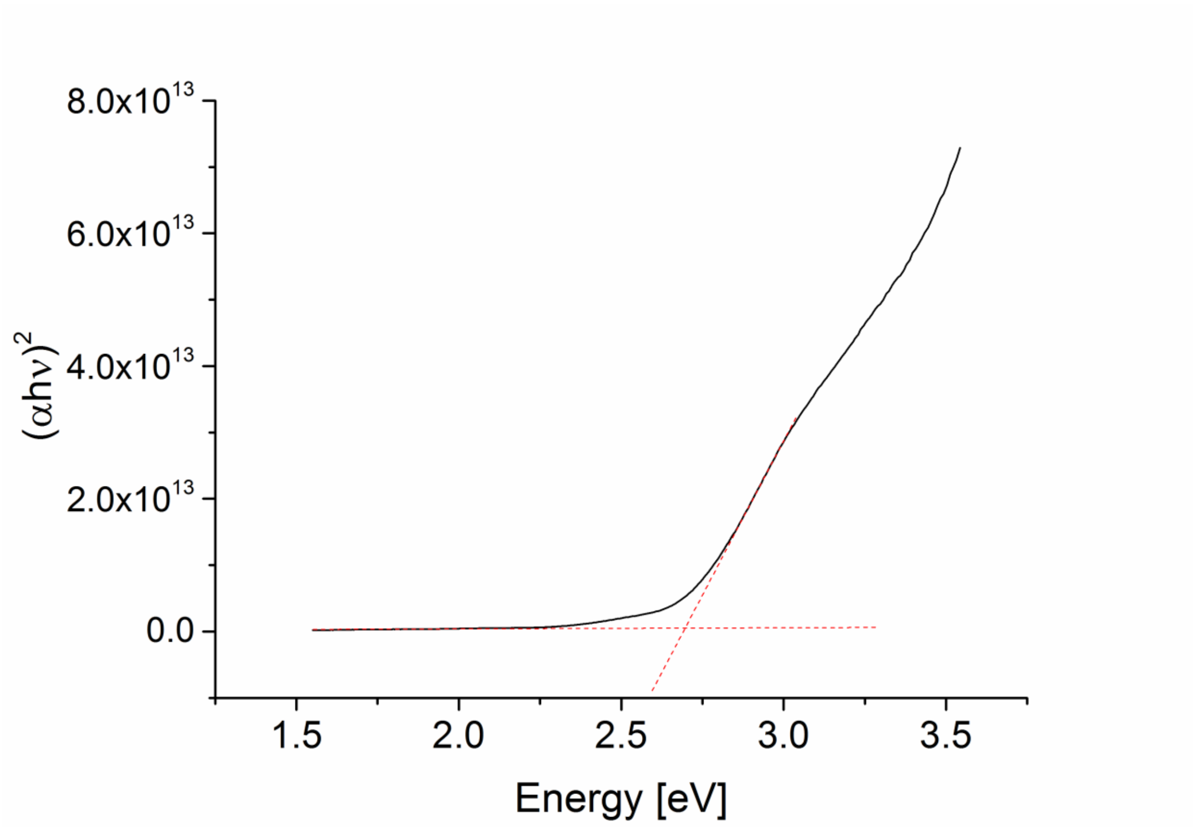

Figure S2 Tauc plot of a $BiFeO_3$ electrode obtained from UV/Vis analysis.[4] From this, a direct optical band gap of about 2.7 eV has been obtained.

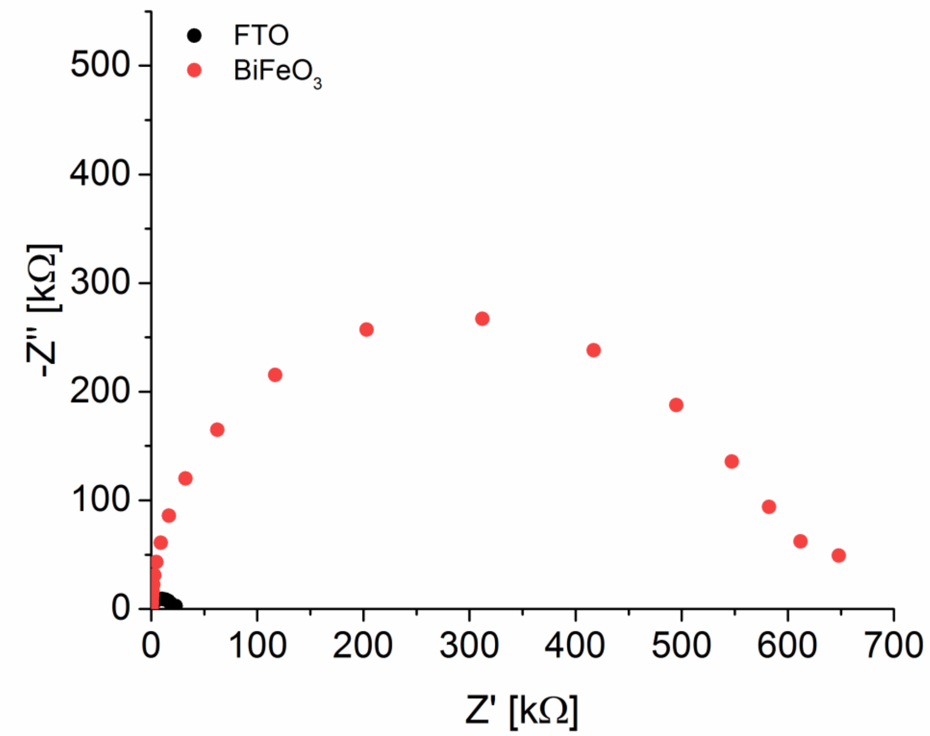

Figure S3 Impedimetric measurements of an pure FTO slide before and after deposition of $BiFeO_3$ in the presence of 2mM ferri-/ferrocyanide at open circuit potential in the dark (OCP: 0.197 V; amplitude: 5 mV, frequency range: 100 kHz – 100 mHz).

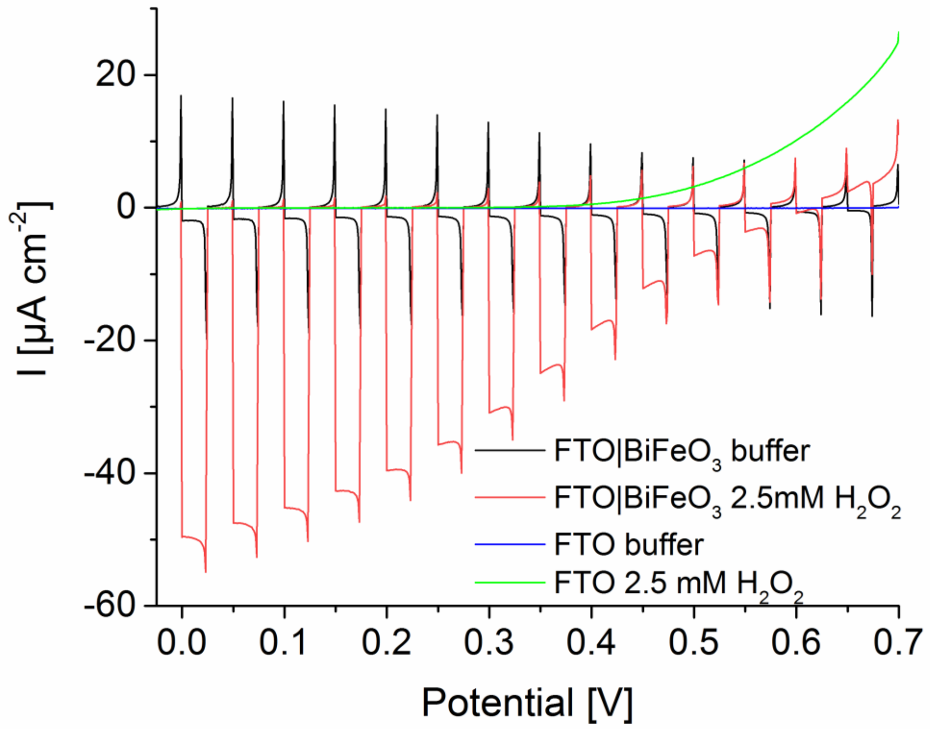

Figure S4 Chopped-light voltammetry of an FTO slide and an FTO|$BiFeO_3$ electrode in the presence and absence of 2.5 mM H_2O_2 (white light source; 100 mW cm^{-2}; potential vs Ag/AgCl, 1 M KCl; 10 mV s^{-1})

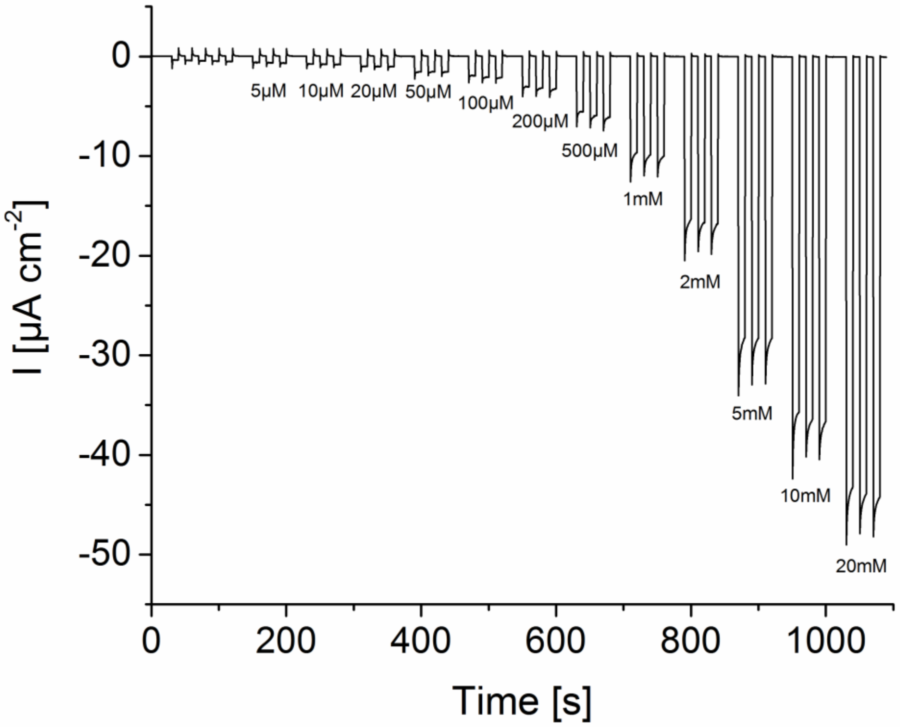

Figure S5 Photoamperometric measurement of an FTO|BiFeO$_3$ electrode with subsequent additions of H_2O_2 (white light source; 100 mW cm^{-2}; 0.2 V vs Ag/AgCl, 1 M KCl).

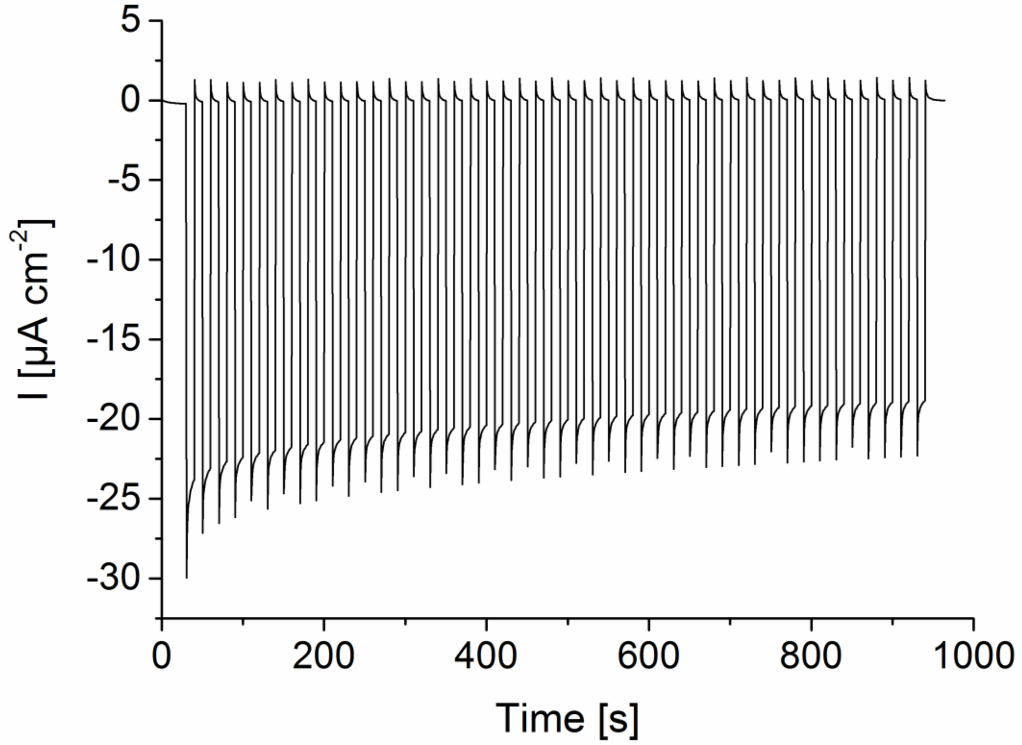

Figure S6 Photoamperometric measurement of an FTO|BiFeO$_3$ electrode in the presence of 2.5 mM H_2O_2 using pulsed illumination over 15 min (white light source; 100 mW cm^{-2}; 0.2 V vs Ag/AgCl, 1 M KCl).

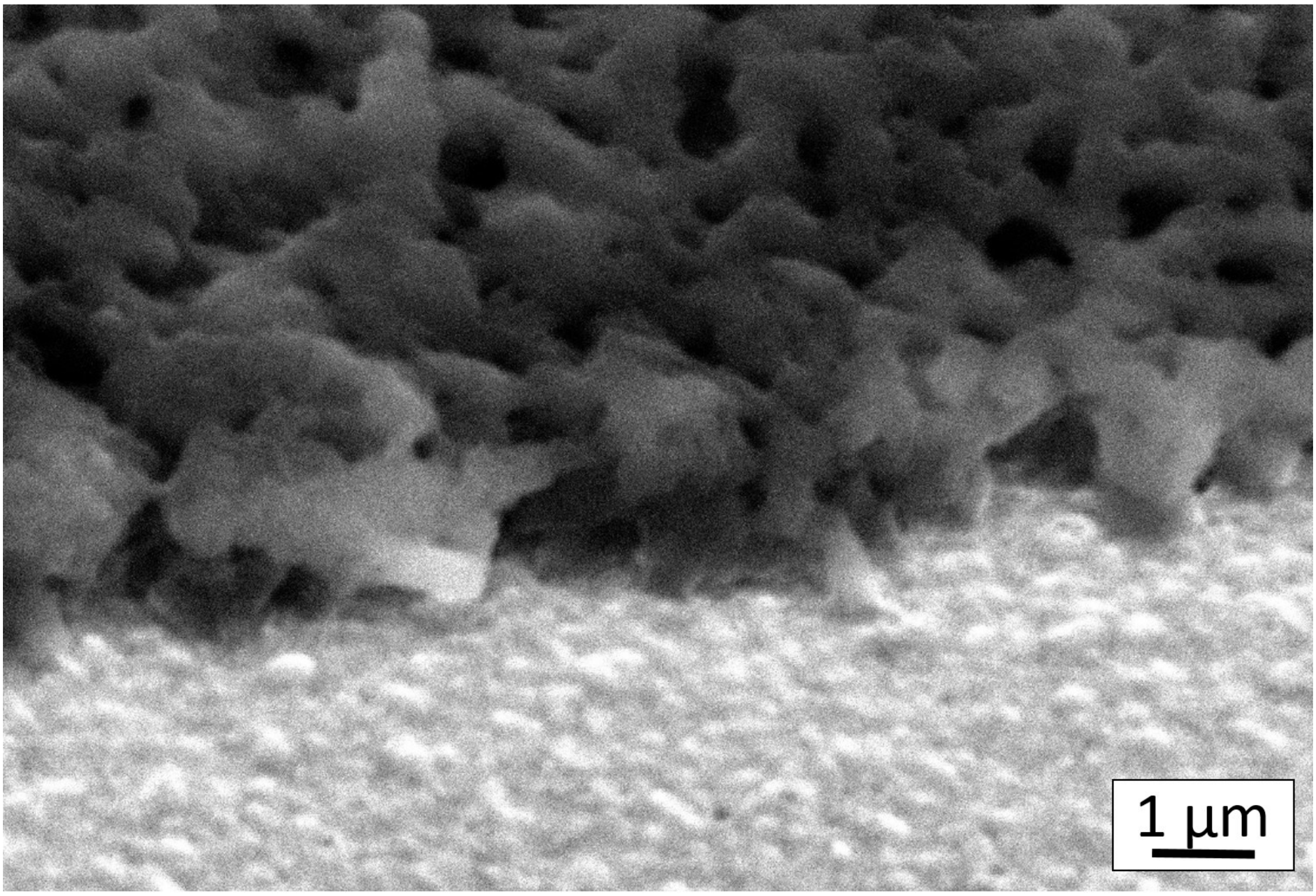

Figure S7 SEM image of a $BiFeO_3$ electrode modified with a polyethyleneimine/GOx mixture under an angle of 45° with a 10.000-fold magnification by applying an acceleration voltage of 15 kV. Therefore, a part of the polymer/enzyme layer was scraped away to create a hard edge, allowing the estimation of the layer height in the dry state. The modification procedure results in a rough polymer/enzyme network with a thickness of about 1.1 µm.

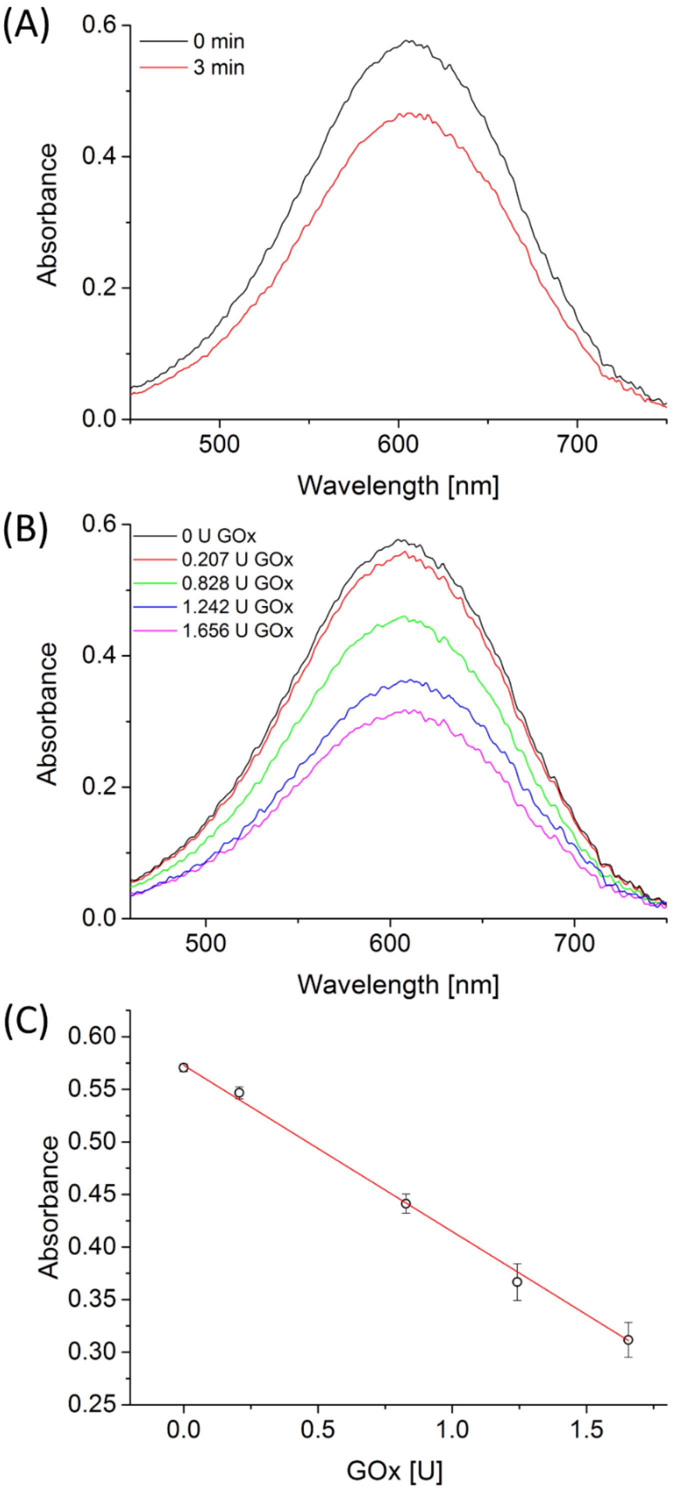

Figure S8 For the determination of the enzymatic activity of GOx bound to the $BiFeO_3$ electrode, an activity assay has been performed using 2,6-dichlorophenolindophenol (DCPIP) as electron acceptor. Therefore, a calibration curve using different GOx activities in solution has been recorded by correlating the DCPIP conversion rate to known GOx activities. The same assay has been carried out on the $BiFeO_3$|GOx electrode, allowing an estimation of the bound GOx activity using the calibration curve. Since the GOx is bound to the electrode and the calibration curve has been recorded with GOx in solution, this only gives an approximation of the immobilized GOx activity.
(A) UV/VIS spectra of 285 µM DCPIP dissolved in 50 mM phosphate buffer pH7 with 100 mM glucose before (black curve) and after reaction with a $BiFeO_3$|GOx electrode for 3 min. (B) UV/VIS spectra of 285 µM DCPIP dissolved in 50 mM phosphate buffer pH7 with 100 mM glucose before (black curve) and after reaction of different GOx activities for 3 min. (C) Calibration curve for GOx in solution, obtained from the UV/VIS experiments at 600 nm. From the calibration curve, the activity of GOx attached to the $BiFeO_3$ electrode has been calculated to be 0.8 ± 0.09 U. All UV/VIS experiments have been performed with a NanoDrop 2000 (Thermo Fisher Scientific Inc.) with a light path length of 1 mm.

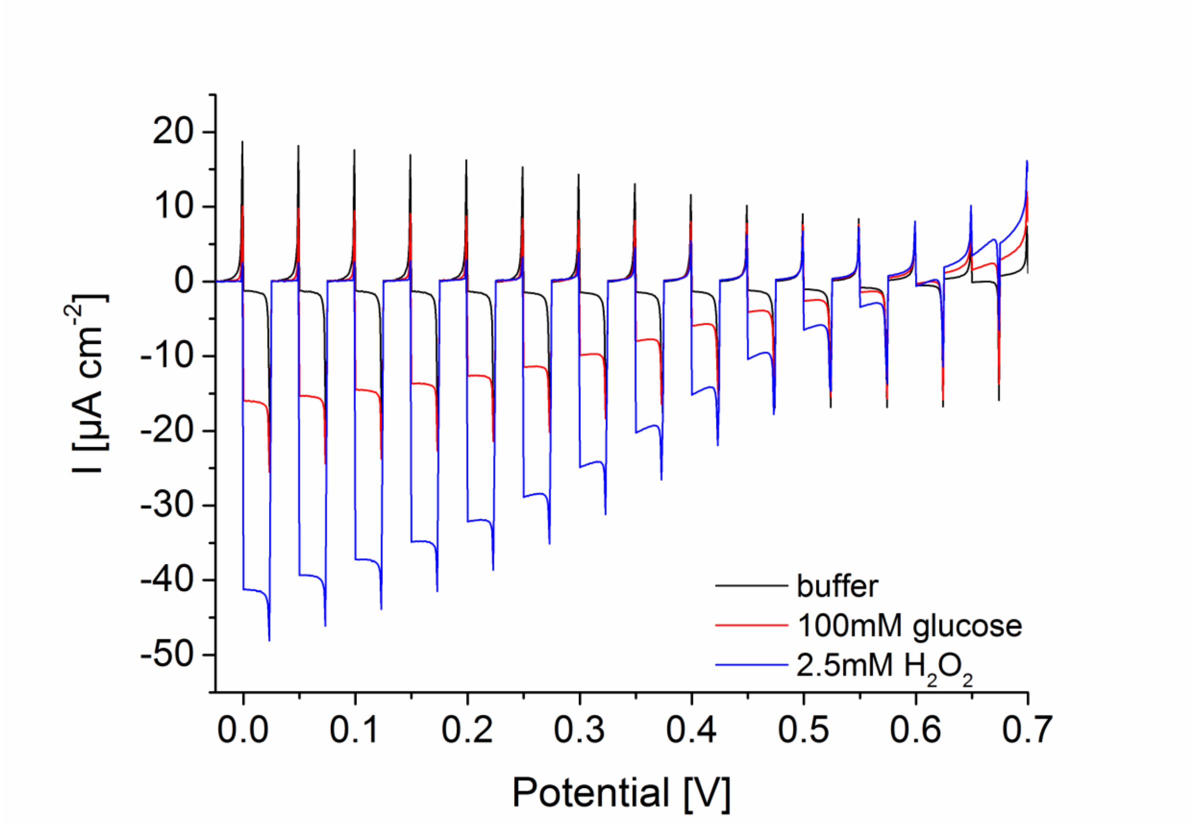

Figure S9 Chopped-light voltammetry of a FTO|$BiFeO_3$|GOx electrode in the presence and absence of 100 mM glucose and 2.5 mM H_2O_2 (white light source; 100 mW cm^{-2}; potential vs Ag/AgCl, 1 M KCl, 10 mVs^{-1}).

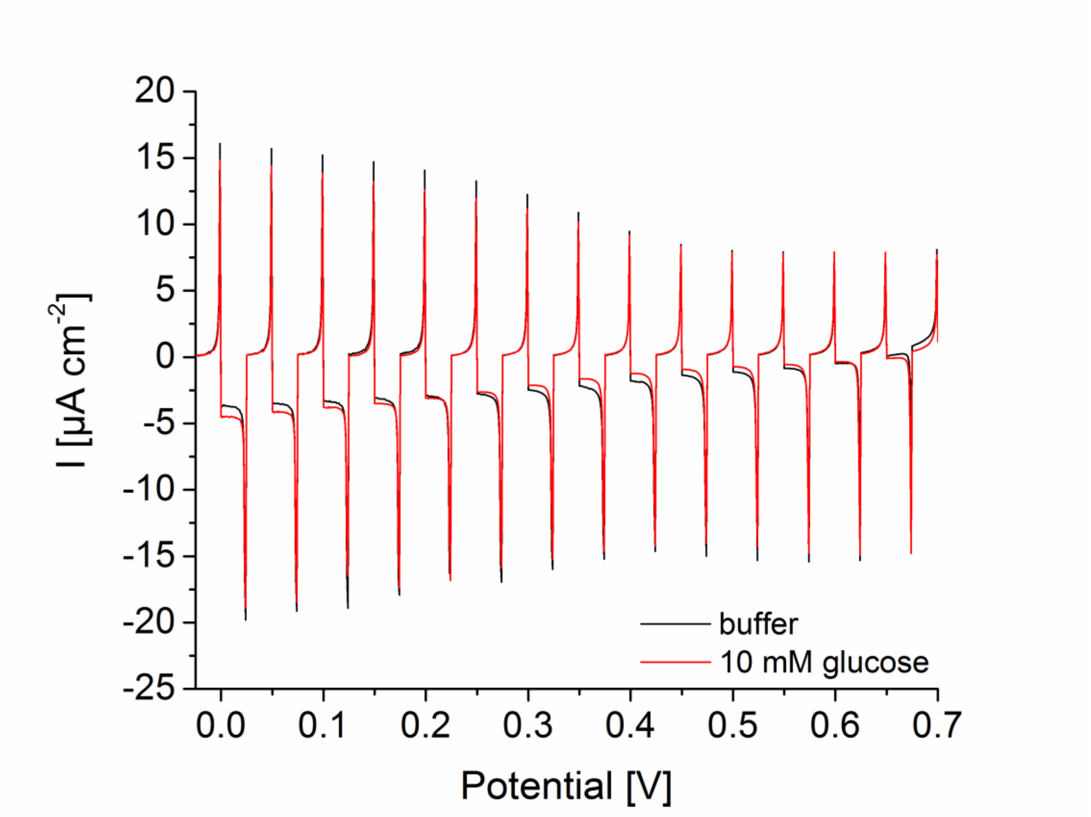

Figure S10 Chopped-light voltammetry of an FTO|$BiFeO_3$ electrode without GOx in the presence and absence of 10 mM glucose (white light source; 100 mW cm^{-2}; potential vs Ag/AgCl, 1 M KCl, 10 mVs^{-1}).

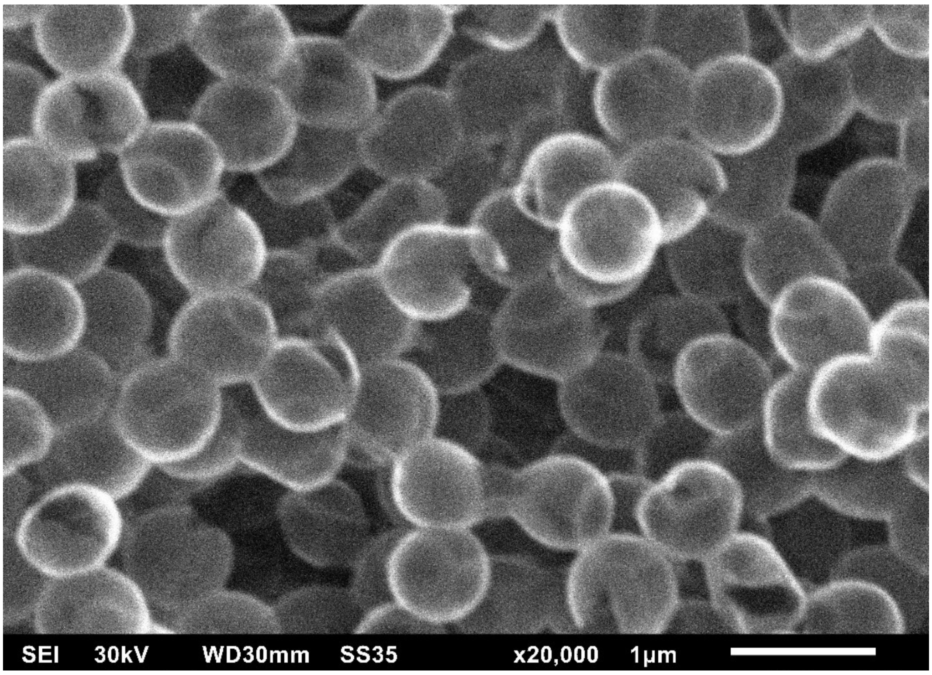

Figure S11 SEM image of an FTO slide modified with IO-TiO_2 using an accelerating voltage of 30 kV and a 20.000-fold magnification. (base electrode of the photobioanode)

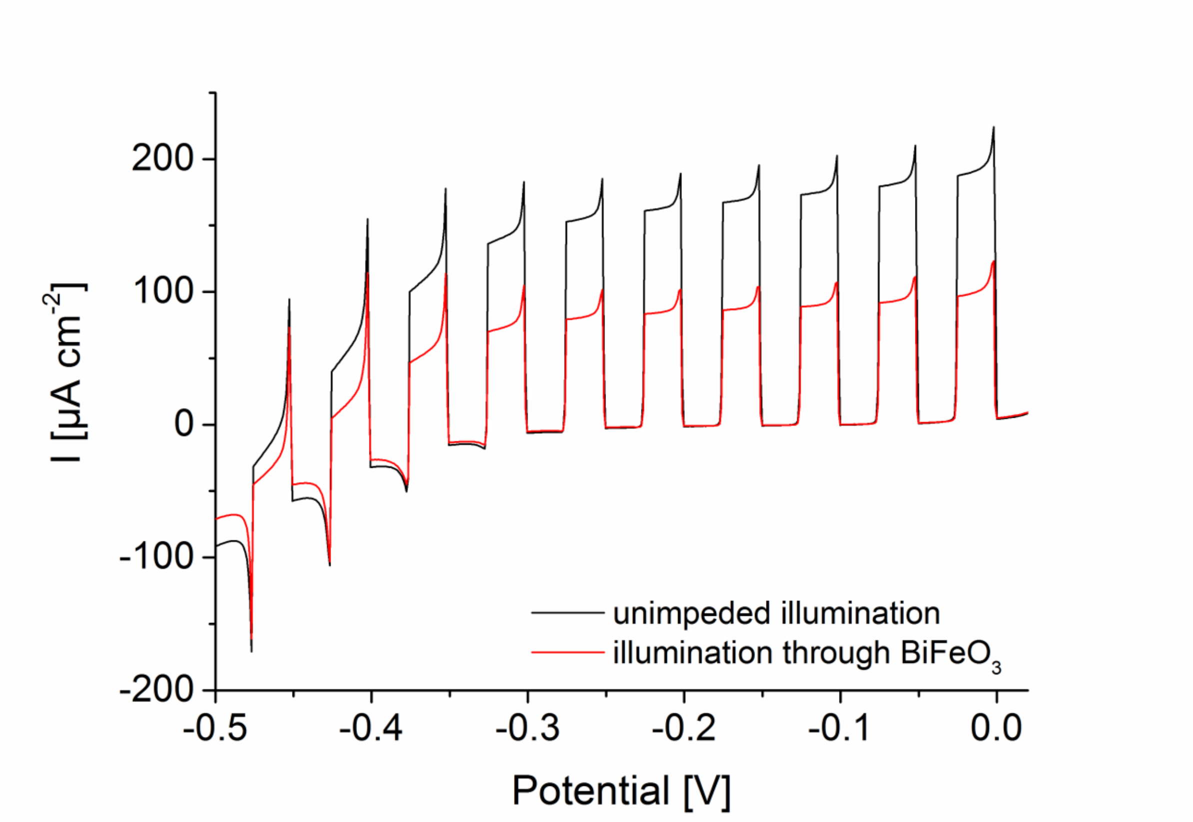

Figure S12 Chopped-light voltammetry of an IO-TiO_2|PbS|P_{Os}|FAD-GDH electrode in the presence of 100 mM glucose using unimpeded illumination and by illumination through FTO|$BiFeO_3$ (white light source; 100 mW cm^{-2}; potential vs Ag/AgCl, 1 M KCl, 10 mVs^{-1}).

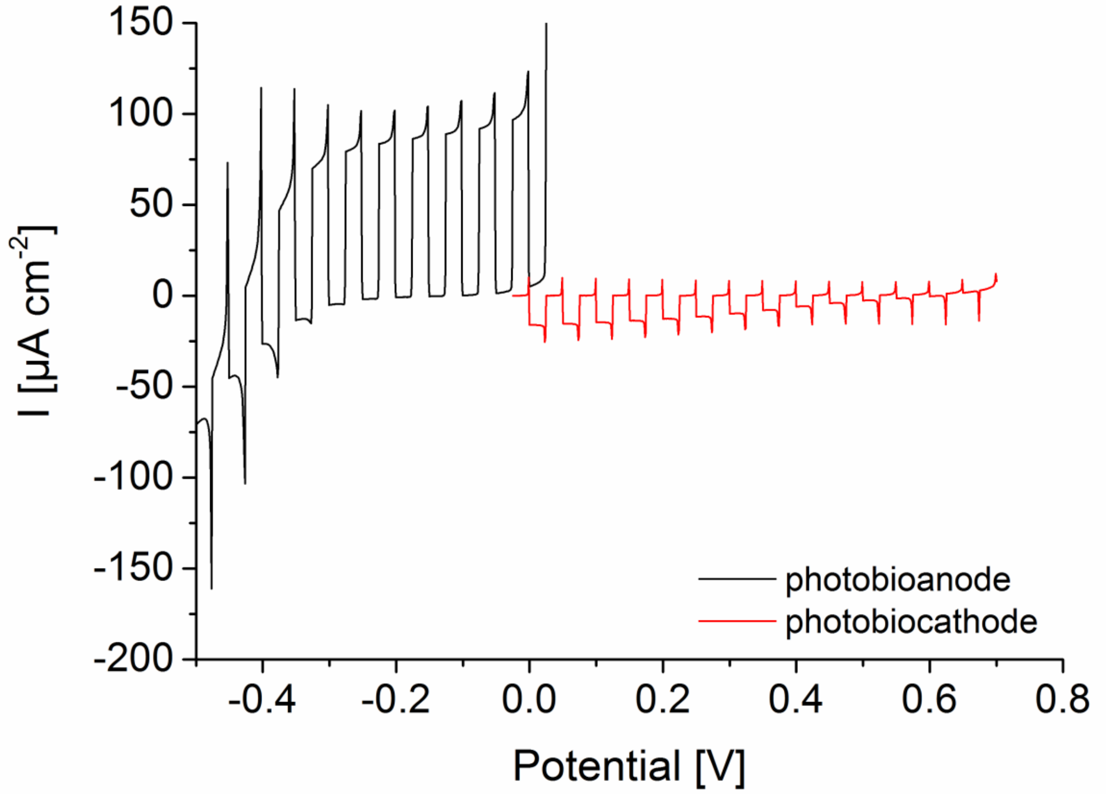

Figure S13 Chopped-light voltammetry of an IO-TiO_2|PbS|P_{Os}|FAD-GDH photobioanode in the presence of 100 mM glucose using illumination through FTO|$BiFeO_3$ and an FTO|$BiFeO_3$|GOx photobiocathode in the presence of 100 mM glucose (white light source; 100 mW cm^{-2}; potential vs Ag/AgCl, 1 M KCl, 10 mVs^{-1}).

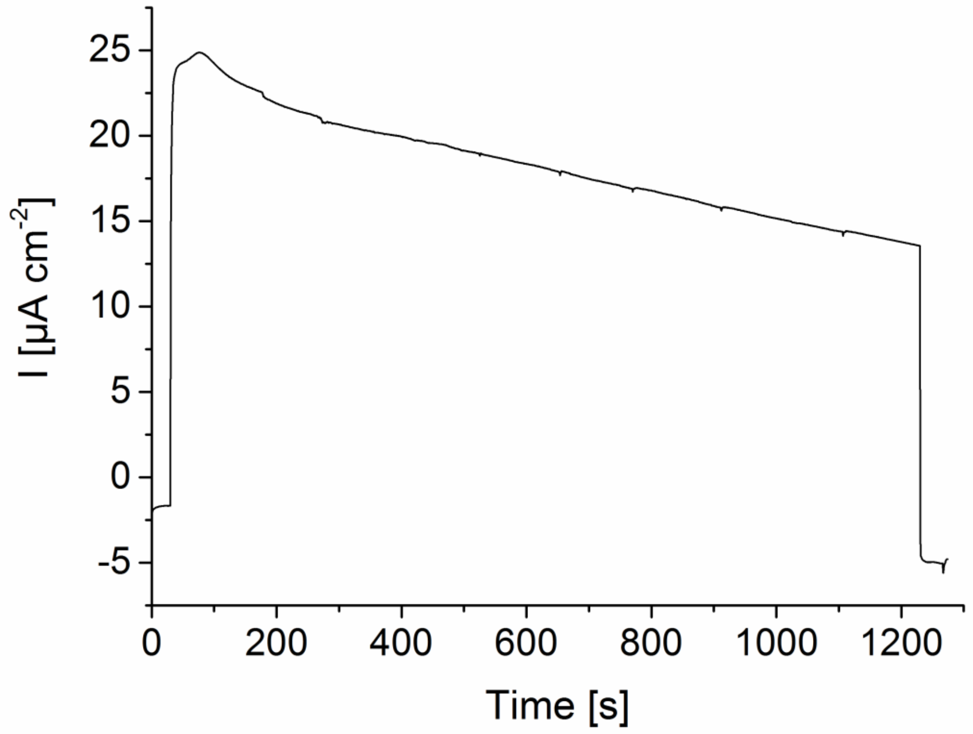

Figure S14 Photoamperometric measurement of a photobioelectrochemical tandem cell consisting of an FTO|$BiFeO_3$|GOx photobiocathode and an IO-TiO_2|PbS|P_{Os}|FAD-GDH photobioanode in the presence of 100 mM glucose using continuous illumination over 20 min (white light source; 100 mW cm^{-2}; -0.6 V).

References

[1] A. Badura, D. Guschin, B. Esper, T. Kothe, S. Neugebauer, W. Schuhmann, M. Rögner, *Electroanalysis* **2008**, *20*, 1043–1047.
[2] P. Yilmaz, D. Yeo, H. Chang, L. Loh, S. Dunn, *Nanotechnology* **2016**, *27*, 345402.
[3] M. Riedel, W. J. Parak, A. Ruff, W. Schuhmann, F. Lisdat, *ACS Catal.* **2018**, *8*, 5212–5220.
[4] J. Tauc, R. Grigorovici, A. Vancu, *Phys. Status Solidi B* **1966**, *15*, 627–637.
